# In vitro modeling of inflammatory bowel diseases using a newly developed immunocompetent colon epithelial monolayer co-culture model

**DOI:** 10.1038/s44385-026-00091-9

**Published:** 2026-06-15

**Authors:** Imen Larafa, Roxana Zogorean, Adrian Buehler, Claudia Günther, Stefan Wirtz, Markus F. Neurath, Oana-Maria Thoma, Maximilian J. Waldner

**Affiliations:** 1 Department of Medicine 1, Friedrich-Alexander-Universität Erlangen-Nürnberg, Erlangen, Germany; 2 Max-Planck-Zentrum für Physik und Medizin, Erlangen, Germany; 3 German Center for Immunotherapy, Deutsches Zentrum Immuntherapie (DZI), University Hospital Erlangen, Friedrich-Alexander-Universität Erlangen-Nürnberg, Erlangen, Germany; 4 Comprehensive Cancer Center Erlangen-EMN (CCC ER-EMN), Erlangen, Germany; 5 Department of Pediatrics and Adolescent Medicine, Friedrich-Alexander-Universität Erlangen-Nürnberg, Erlangen, Germany; 6 Medical Immunology Campus Erlangen, FAU Erlangen-Nürnberg, Erlangen, Germany; 7 Erlangen Graduate School in Advanced Optical Technologies (SAOT), Friedrich-Alexander-Universität Erlangen-Nürnberg, Erlangen, Germany

**Keywords:** Gastroenterology, Immunology, Microbiology

## Abstract

Clinical treatment of inflammatory bowel diseases (IBD) remains challenging due to the complex interplay between the epithelial barrier, immune system, and gut microbiota. While in vitro models are pivotal for studying barrier dysfunction, developing a standardized and functionally relevant system for IBD remains challenging. To overcome this, we established an immunocompetent murine colon epithelium monolayer to model IBD-like conditions. Colons from wild-type mice were digested into single cells and seeded onto Matrigel-coated transwells. Within seven days, monolayers showed strong barrier properties and displayed epithelial cell lineage, including goblet, stem, and enteroendocrine cells. However, exposure to pro-inflammatory cytokines as well as infection with pathogenic bacteria, including *Clostridium rodentium and Salmonella Typhimurium*, disrupted epithelial integrity. To better reflect the in vivo state, polarized T cells and macrophages were co-cultured with the epithelium. Pro-inflammatory Th1 and Th17 cells impaired barrier function, while M0 and M2 macrophages maintained it, representing both homeostatic and disrupted conditions of the gut. Upon *Salmonella Typhimurium* infection, M1 macrophages produced IFN-γ, and M2 macrophages secreted IL-10 and enhanced ZO-1 expression. Overall, our model presents a promising platform to study epithelial barrier dysfunction, immune-epithelial cross-talk, and host-pathogen interactions, offering valuable insights into IBD mechanisms and potential therapeutic approaches.

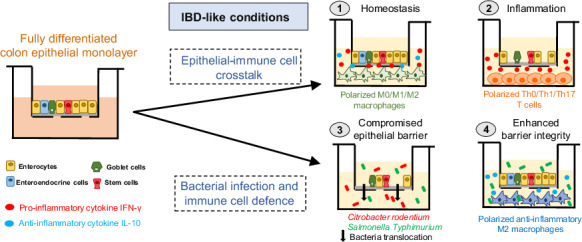

## Introduction

Inflammatory bowel diseases (IBD), including Crohn’s disease and ulcerative colitis, are persistent inflammatory conditions affecting the gastrointestinal tract. The prevalence of IBD has seen a notable increase over the past few decades, which now reaches an estimated 6 to 8 million individuals globally^1,2^. The onset and progression of IBD arise from various factors such as genetics, immune modulation, environmental conditions and microbiota, all of which exert an influence on the intestinal epithelium in the gut^3,4^. Particularly, the gut is a highly organized dynamic organ, primarily composed of the intestinal epithelial layer and the lamina propria, which contains the immune system. In homeostasis, the intestinal barrier serves as a protective physical barrier between luminal microbiota and the mucosal immune system in order to maintain internal balance and protect from external threats^5,6^. In inflammatory conditions, the intestinal barrier is disrupted, leading to increased gut permeability and microbial infiltration. This triggers an overreaction of the immune system, resulting in the excessive production of pro-inflammatory mediators such as cytokines and chemokines. Repetitive cycles of bacterial infiltration, immune activation and tissue damage trigger gut inflammation and amplify inflammatory responses, eventually disrupting the balance of the gut environment^7,8^.

To gain a deeper comprehension of the pathophysiology of IBD and elucidate its mechanisms, diverse mouse models have played a pivotal role in preclinical research and drug development, amongst others. These include chemically induced models, employing compounds such as dextran sulfate sodium (DSS), trinitrobenzene sulfonic acid (TNBS), and oxazolone. Furthermore, various genetically engineered models have been used to study the development of spontaneous intestinal inflammation, including mice engineered to express elevated TNF-α, and those with gene deficiencies such as I-kappa-B kinase gamma (Iκκ-γ) and interleukin (IL)-10 models. Other colitis models using anti-CD40 or the adoptive transfer of naïve CD4+ T cells are also commonly used to study how immune cells contribute to the onset of inflammation^9–11^. Lastly, given the key role of microbiota in IBD, bacteria-induced preclinical mouse models have been highly employed. For example, *Citrobacter rodentium* infection in mice resulted in acute intestinal inflammation and compromised barrier function. Also, *Salmonella Typhimurium* infection in germ-free mice led to severe enterocolitis. Other bacteria could also be used, such as Helicobacter hepaticus, which triggers colitis in genetically susceptible or immunodeficient strains^12^.

Animal models offer a major advantage due to their ability to reproduce a variety of features similar to human disease. In addition, they are essential for understanding the underlying mechanisms and for testing new therapeutic candidates that target specific elements involved in disease progression^9,13^. However, using animals to model disease may not only fail to fully represent the natural course of the disease, but also raise ethical concerns^14,15^.

Lately, in vitro models have emerged as powerful tools that allow the study of specific cell types or processes in a more controlled environment^16^. For example, organoid cultures have revolutionized the understanding of the structural and biological complexity of an organ, due to their capability to mimic in vivo-like characteristics in vitro. They can be derived from pluripotent or tissue-resident stem cells from healthy or diseased tissues, making them suitable for modeling a wide range of healthy and diseased conditions^17^. Furthermore, the development of organ-on-a-chip systems is another bioengineering approach that is gaining interest as a next-generation experimental tool. This technique combines biology with advanced microfluidic platforms that recapitulate tissue-specific functions, which closely resemble human physiology^18^. In vitro systems are therefore valuable not only for investigating the underlying mechanisms of disease but also potentially bridge the gap between conventional cell culture and animal model systems^16,19^. In the field of gastroenterology, traditional models rely on immortalized tumor cell lines, such as the Caco-2 or HT-29. These in vitro 2D cultures lack epithelial lineage diversity and are characterized by aberrant cell proliferation that does not reflect the homeostatic in vivo epithelial structure^20^. On the other hand, 3D in vitro models using intestinal organoids are better at reproducing organ physiology and can be used to study a variety of physiological mechanisms, including cell-to-cell communication and host-pathogen interaction^21^. Particularly, intestinal organoids are characterized by the self-renewal of stem cells and the differentiation towards major intestinal lineages^22,23^.

Despite the numerous advantages that organoids offer, their enclosed structure poses a significant limitation. The epithelial layer of the organoid is situated on its inner surface, making it challenging to access the appropriate compartment. For example, host-pathogen interactions require access to the lumen-facing side, while metabolites are best delivered to the basal side. Additionally, dead cells can accumulate in the lumen, thus necessitating the splitting and reseeding of organoids^24^. Therefore, relatively complicated approaches are the go-to methods for accessing the epithelium. Examples of these approaches include microinjection and reversing organoid polarity^25^.

In this regard, 2D monolayer cultures present distinct advantages by enabling direct access to the epithelial layer and facilitating the investigation of the intestinal barrier function and cellular interactions^26,27^. Moreover, they are useful for exploring cell signaling dynamics, barrier permeability and polarity, as they enable access to both the apical and basolateral surfaces of the epithelium^20,26^.

Here, we present the development and functional evaluation of a novel murine colonic epithelial monolayer model that incorporates innate and adaptive immune cells. The monolayers were subjected to three distinct applications closely resembling aspects of IBD pathology. Firstly, the model was stimulated with various pro-inflammatory cytokines, similar to what is typical in IBD patients. Secondly, we investigated the effect of polarized immune T cells and macrophages on barrier integrity to better mimic the interactions between intestinal epithelial cells and the immune system in inflammatory settings. Then, we conducted infection studies using *Salmonella Typhimurium* and *Citrobacter rodentium* to understand the host-pathogen interactions. Lastly, we explored the impact of macrophages on bacterial infection, shedding light on the interplay between the innate immune system and the epithelial layer during bacterial infection in the context of IBD.

Our model represents a reliable experimental tool that can be used to support in vivo experiments, which can potentially lead to a better understanding of IBD and the development of new treatments in the future.

## Results

### In vitro colon epithelial monolayers display in vivo-like characteristics

**Fig. 1:**
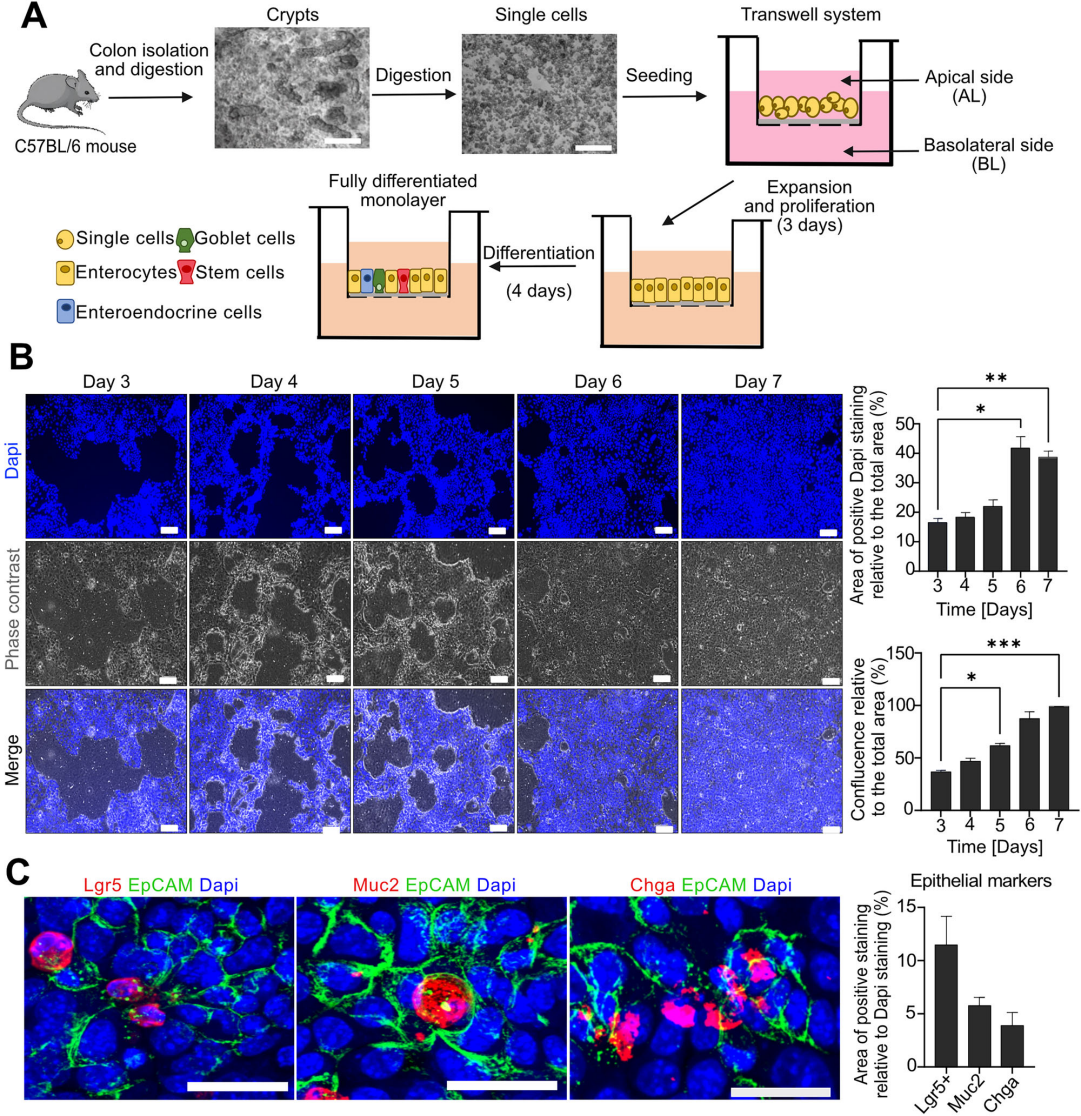
Development of mouse colon epithelial monolayer cultures using a transwell system. **A** Schematic representation of the experimental setup employed: Crypts were isolated from the mouse colon and were digested into single cells. Transwell systems were coated with a thin layer of Matrigel, and the cells were then plated in the seeding medium (for 3 days), followed by differentiation medium (starting day 3) and incubated until fully grown. **B** Immunohistochemistry staining of the monolayers collected from day 3 to day 7 of culture (scale bar = 100 µm). Nuclei were stained with Dapi (blue), and quantification was done with ImageJ software by calculating the area of the monolayer relative to the total area (*n* = 3 samples per group, experiment repeated at least 2 times). **C** Immunohistochemistry staining for major epithelial markers of the colon cultures. Cell nuclei were counterstained with Dapi (scale bar = 20 µm), and quantification was done by calculating the area of positive staining relative to Dapi staining (*n* ≥ 3 samples per group, pooled from 3 independent experiments). The data show the mean ± SEM, * adjusted *p* ≤ 0.05, ** adjusted *p* ≤ 0.01, *** adjusted *p* ≤ 0.001. Statistical differences were calculated using one-way ANOVA with Dunnett´s multiple comparisons test.

To establish monolayer cell cultures, crypts were released from the colon and digested in order to isolate single cells, which were then carefully seeded onto a Matrigel-coated transwell system (Fig. 1A and Supplementary Fig. 1). Initially, the cultures were supplemented with seeding media to optimize stem cell enrichment and promote proliferation. Subsequently, differentiation media were added to promote cell differentiation. Monolayer growth was then assessed by immunohistochemistry (Supplementary Fig. 2). After 3 days of culture, the cells attached to the Matrigel-coated insert, with 36.69 ± 1.55% of the insert surface being covered. By day 7, the cells continued to expand and reached 98.67 ± 0.41% of confluence (Fig. 1B). Also, the area of positive staining for the proliferation marker Ki67 decreased from day 3 to day 7, indicating successful epithelial cell growth and demonstrating the shift toward differentiation (Supplementary Fig. 3A, B).

Given the fact that the colon epithelium consists of various cell types, such as epithelial cells, goblet cells, enteroendocrine cells, and stem cells, we aimed to characterize the monolayer to assess the presence of these cells. Immunohistochemistry staining (Fig. 1C and Supplementary Fig. 3A, B) showed the expression of epithelial cell marker EpCAM, goblet cell marker mucin 2 (Muc2) and enteroendocrine cell marker chromogranin A (Chga). Notably, the identification of stem cells, evidenced by Lgr5 positivity (11.40 ± 2.71% of the total cell lineage), validated regenerative potential within the monolayers. Taken together, these findings suggest successful growth and differentiation of the monolayers, with expression of key markers indicative of major colon cell lineages.

### In vitro-generated colon epithelial monolayers show robust barrier function

**Fig. 2:**
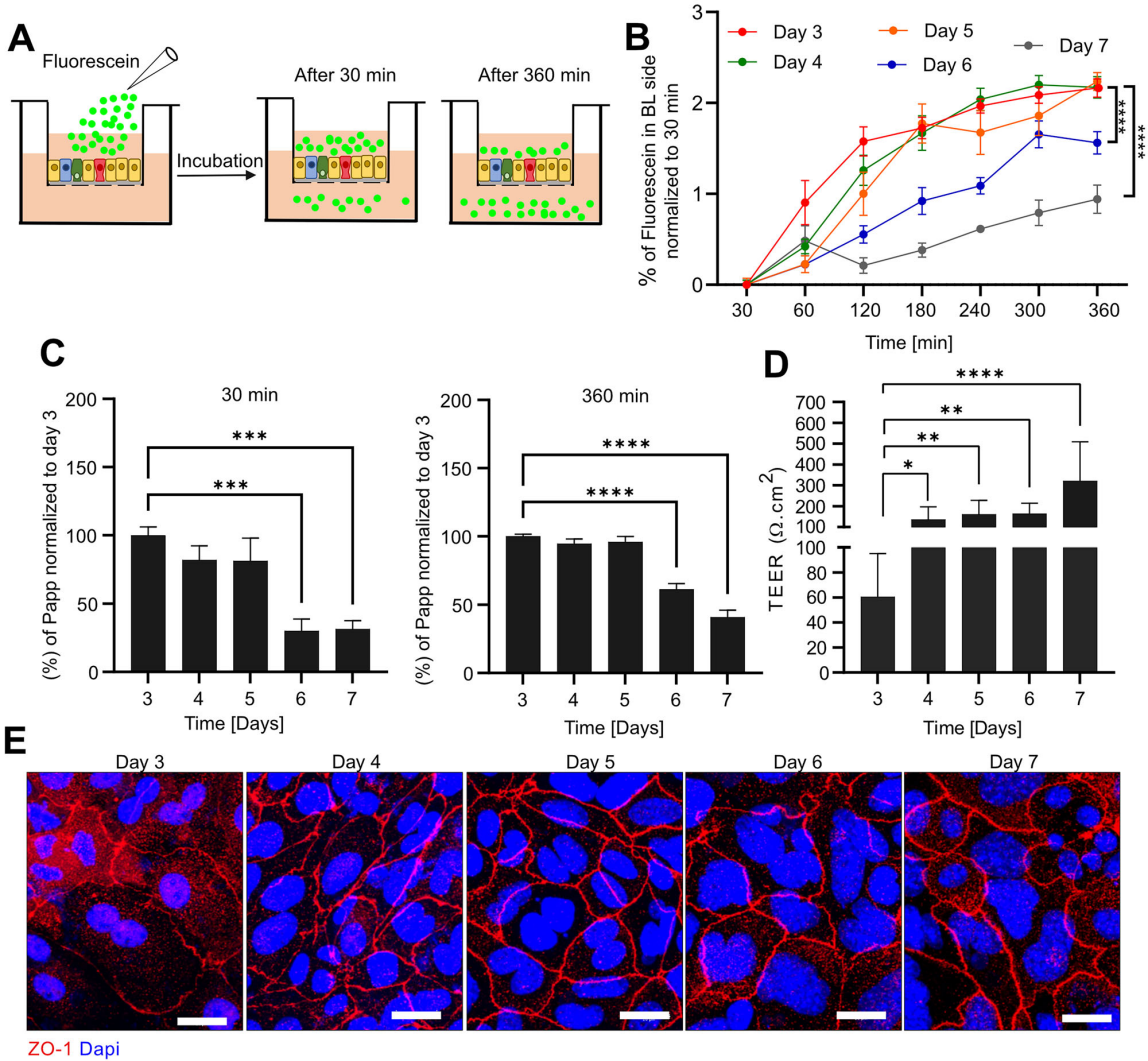
Colon epithelial monolayer cultures show robust barrier integrity characteristics. **A** Schematic representation of the fluorescein permeability assay: Fluorescein was added to the apical side of the monolayer, and **B** was quantified in the basolateral side after different time points of incubation (*n* = 6 samples per group, pooled from 2 independent experiments). **C** Daily quantification of apparent permeability (Papp) values from day 3 to day 7 during monolayer formation, after 30–360 min of incubation with fluorescein (*n* = 6 samples per group pooled from 2 independent experiments). **D** Barrier integrity was assessed by transepithelial electrical resistance measurements (TEER) (*n* ≥ 10 samples per group, pooled from 2 independent experiments). **E** Immunohistochemistry staining of tight junction protein ZO-1 in monolayers collected from day 3 to day 7 of culture (*n* ≥ 2 samples per group, except for day 7, *n* ≥ 4 pooled from 2 independent experiments). Nuclei were stained with Dapi (blue) (scale bar = 20 µm). Graphs show the mean ± SEM, * adjusted *p* ≤ 0.05, ** adjusted *p* ≤ 0.01, *** adjusted *p* ≤ 0.001. Statistical differences were calculated using one-way ANOVA corrected with Dunnett´s multiple comparisons test and with two-way ANOVA with Šídák’s multiple comparisons test for grouped analysis.

Intestinal permeability plays an important role in the gut by regulating the passage of substances through the intestinal epithelium. Increased permeability levels have been associated with various gastrointestinal disorders, including IBD. Thus, ensuring the maintenance of appropriate permeability levels is essential for normal physiological function^28–30^. To evaluate the barrier permeability during the generation of epithelial monolayers (day 3 to day 7 post-seeding), we first used a fluorescein assay. Fluorescein was added to the apical side and its translocation to the basolateral side was measured starting from 30 min until 6 h of incubation (Fig. 2A). As the monolayer was getting formed, significantly less fluorescein passed through the epithelial barrier going from 2.16 ± 0.1% at day 3 to 0.94 ± 0.15% at day 7, after 6 h (Fig. 2B). This was also reflected by Papp evaluation, where the monolayer was highly permeable for the first 5 days, but became significantly less permeable by day 7 (Fig. 2C). Furthermore, TEER measurements of the monolayers revealed a significant increase from day 3 to day 7 (from 60.58 ± 28.07 to 321.1 ± 38.38 Ω∙cm^2^), suggesting that the monolayer´s tightness and integrity have been increasing overtime while the epithelium was also becoming confluent (Fig. 2D). The strong barrier integrity was also confirmed by immunohistochemistry staining, which showed that the monolayer developed tight junctions between the cells, as shown by the expression of the tight junction protein ZO-1 (Fig. 2E and Supplementary Movie 1). Collectively, we confirmed that the developed epithelial monolayer model displays decreased permeability and tight junction markers as it forms, which closely mimic the healthy epithelium of the in vivo colon. Interestingly, when we compared our monolayer model to previously established methods based on organoid cultures, we observed that our protocol achieved a fully confluent monolayer in just 7 days, whereas the conventional method that started from 3D organoids required around 21 days to reach full confluence (Supplementary Fig. 4A, B). Moreover, our system exhibited higher TEER values compared to conventional monolayers that start from 3D organoids. This indicates that this model not only reaches confluence faster but also establishes a stronger barrier function in a shorter time frame. Most importantly, the monolayer could be maintained in culture for up to 22 days by daily changing the medium, further demonstrating the stability of the setup.

### Exposure of colon monolayers to pro-inflammatory cytokines reduces barrier integrity

**Fig. 3:**
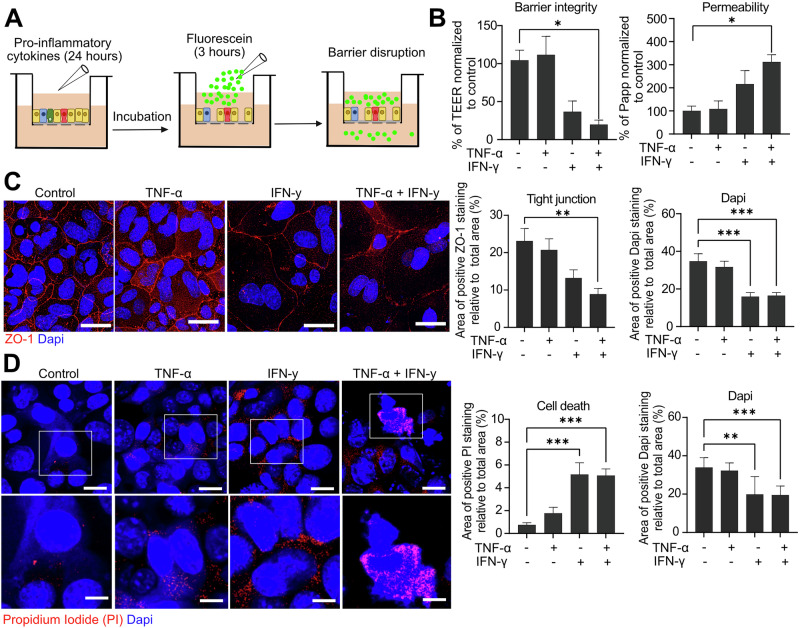
Enhanced barrier disruption and epithelial cell death induced by pro-inflammatory cytokines. **A** Colon monolayers were stimulated with TNF-α, IFN-γ or a combination of both cytokines for 24 h. **B** Barrier integrity was assessed by permeability assay and TEER measurements (*n* = 4 samples per group). **C** Immunohistological stainings for tight junction protein ZO-1 (red), as well as Dapi (blue) for nuclei (scale bar = 30 µm). Quantification was done with ImageJ software by calculating the areas of positive ZO-1 staining and Dapi positive staining relative to the total area (*n* ≥ 4 samples per group, pooled from 2 independent experiments). **D** Immunohistological stainings for cell death using propidium iodide PI (red), as well as Dapi (blue) for nuclei (scale bar = 10 µm and 5 µm, respectively). Quantification was done with ImageJ software by calculating the areas of positive PI staining and Dapi positive staining relative to the total area (*n* = 6 samples per group, pooled from 2 independent experiments). Graphs show the mean ± SEM, * adjusted *p* ≤ 0.05, ** adjusted *p* ≤ 0.01, *** adjusted *p* ≤ 0.001. Significant differences were observed by using one-way ANOVA corrected with Dunnett´s multiple comparison test.

In IBD, TNF-α and IFN-γ play significant roles in the regulation of intestinal barrier integrity^28,31^. Both cytokines have been associated with a disruption of the barrier function of the intestinal epithelium, resulting in an increase in intestinal epithelial cell death^1,29^. Thus, we evaluated the effect of TNF-α and IFN-γ on the epithelial integrity of colon monolayers. Epithelial monolayers were incubated with TNF-α and IFN-γ for 24 h (Fig. 3A). We then evaluated barrier integrity after stimulation, using TEER measurements and fluorescein permeability assay (Fig. 3B). Interestingly, the treatment with TNF-α had no discernible effect on permeability or TEER values. However, IFN-γ induced the disruption of the monolayer, showed by an increase in percentage of normalized apparent permeability Papp (216.20 ± 58.44%) in treated ones and a decrease in TEER (36.60 ± 14.22%) compared to the control. Notably, exposure to a combination of TNF-α and IFN-γ resulted in the most significant barrier disruption, with a significant decrease in normalized TEER (19.80 ± 5.91%) and a significant increase in normalized permeability (311.90 ± 31.81%) compared to untreated controls. Barrier damage was also observed by evaluating the tight junction protein expression ZO-1, which decreased after treatment with IFN-γ alone (from 20.12 ± 4.3 7to 13.18 ± 4.37%) or in combination of TNF-α and IFN-γ (from 23.12 ± 3.85% to 8.87 ± 3.85%), as shown in Fig. 3C. Another important marker of IBD is epithelial cell death that can be triggered by dysregulation of pathways within the intestinal epithelium^30,32^. To address this matter, we assessed epithelial cell death upon cytokine stimulation by performing PI staining (Fig. 3D). Unlike TNF-α, which had no significant effect, IFN-γ treatment resulted in increased cell death (from 0.17 ± 0.93% to 5.15 ± 0.93%). Also, its combination with TNF-α led to the high cell death rates within the monolayer (5.072 ± 0.93%). These results revealed that our monolayer system is responsive to TNF-α and IFN-γ and could be used to evaluate the effect of other pro-inflammatory molecules.

### Establishment of immunocompetent co-cultures of monolayers with polarized T cells show compromised barrier integrity

**Fig. 4:**
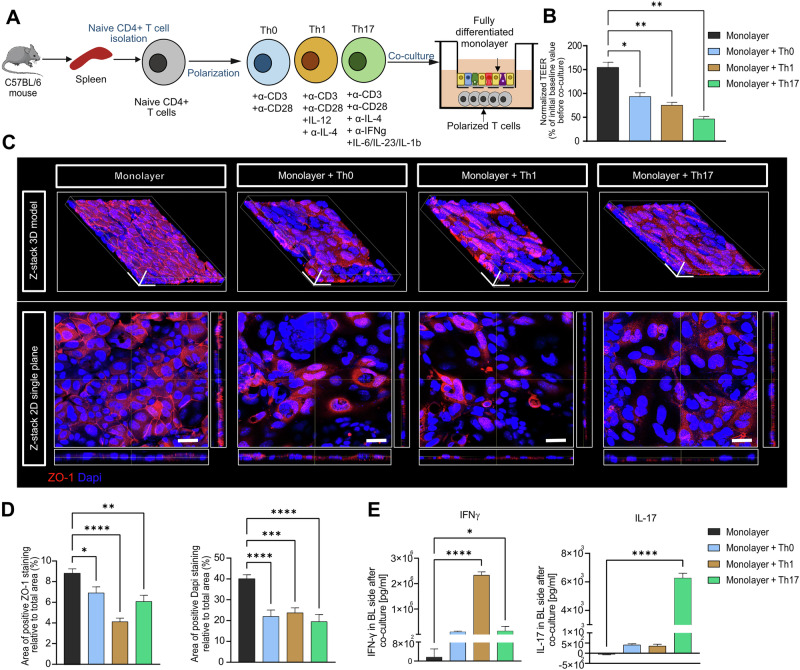
Co-culture of polarized T cells with colon monolayers induced barrier damage. **A** Schematic representation of the experimental setup employed: CD4+ CD25- T cells were isolated from the mouse spleen and were polarized into Th0, Th1 and Th17 cells for 3 days. After that, the medium was changed, and the cells were put in co-culture with a fully differentiated monolayer for 24 h. **B** Barrier integrity was assessed by TEER measurements (*n* = 4 samples per group, experiment repeated at least 2 times). **C** Immunohistological stainings for tight junction protein ZO-1 (red), as well as Dapi (blue) for nuclei. The images show 3D Z-stack confocal imaging (scale bar: *x*, *y*, and *z* = 100 μm) and their 2D orthogonal average intensity projections (scale bar: xy, xz and yz = 30 μm). **D** Quantification was done with ImageJ software by calculating the areas of positive ZO-1 staining and Dapi positive staining relative to the total area (*n* ≥ 10 samples per group, pooled from 3 independent experiments). **E** Enzyme-linked immunosorbent assay (ELISA) measurements show IFN-γ and IL-17 cytokine production in the basolateral side of the monolayer (*n* ≥ 10 samples per group, pooled from 3 independent experiments). Graphs show the mean ± SEM, * adjusted *p* ≤ 0.05, ** adjusted *p* ≤ 0.01, *** adjusted *p* ≤ 0.001. Significant differences were observed by using one-way ANOVA corrected with Dunnett´s multiple comparison test.

Adaptive immune cells, notably T cells, play a critical role in the pathogenesis of IBD through their impact on epithelial barrier integrity^33^. During inflammation, the exaggerated reaction of activated T cells leads to the production of cytokines that disrupt the tight junction and therefore induce epithelial barrier loss and cell death^34–36^. Thus, in order to model this process and further investigate the impact of T cells on barrier integrity, we first isolated naïve CD4+ CD25- T cells from the spleen of wild-type mice and polarized them into Th0, Th1, and Th17 subsets. The cells were then put in co-culture with a fully differentiated monolayer for 24 h (Fig. 4A). Interestingly, Th17 cells exhibited the most pronounced effect in disrupting the epithelial barrier, as shown by reduced TEER (from 157.4 ± 13.16% in control monolayers compared to 46.65 ± 13.27% in monolayers co-cultured with Th17 cells). In comparison, Th1 cells led to significant reduction of TEER to 75.20 ± 6.93%, while Th0 cells had the least impact (93.60 ± 13.16%, Fig. 4B). Tight junction staining of ZO-1 (Fig. 4C, D) further confirmed these findings, where a significant decrease in protein levels was predominant for Th17 and Th1 co-cultures (6.07 ± 0.72% and 4.11 ± 0.72% respectively, compared to 8.81 ± 0.72% in control monolayers). This barrier disruption may be attributed to cytokine secretion, as Th17 cells produced IL-17a (6271 ± 249 pg/ml), which has been previously implicated in barrier damage (Fig. 4E)^6^. Similarly, Th1 cells expressed IFN-γ (233.45 ± 47.15 ng/ml), which also contributed to epithelial integrity loss.

### Immunocompetent co-cultures of monolayers with polarized macrophages mimic homeostasis

**Fig. 5:**
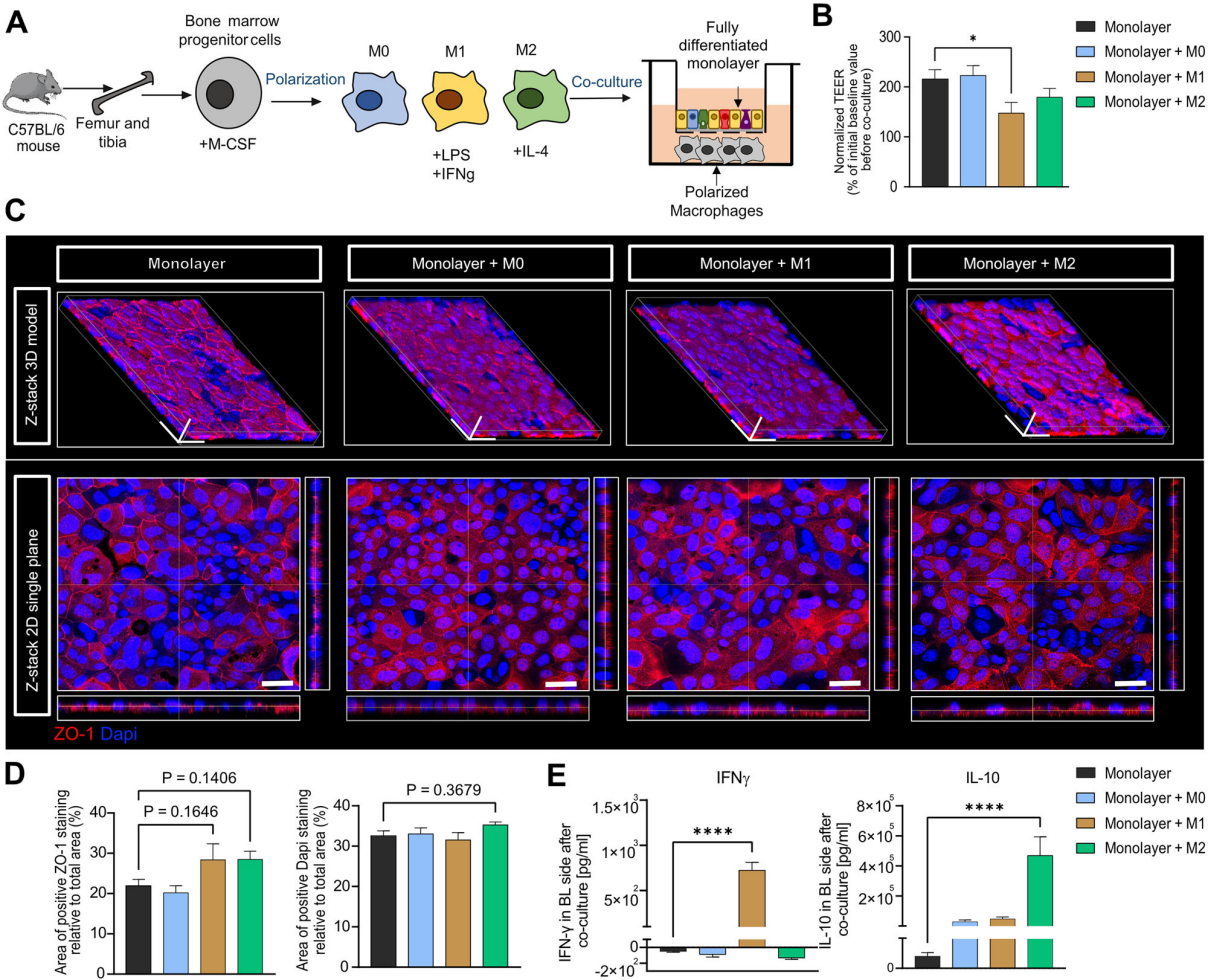
Co-culture of polarized macrophages with colon monolayers mimics homeostatic condition. **A** Schematic representation of the experimental setup employed: Bone marrow progenitor cells were isolated from the mouse femur and tibia and were polarized into M0, M1, and M2 macrophages for 9 days. After that, the medium was changed, and the cells were put in co-culture with a fully differentiated monolayer for 24 h. **B** Barrier integrity was assessed by TEER measurements (*n* ≥ 15 samples per group, pooled from 5 independent experiments). **C** Immunohistological stainings for tight junction protein ZO-1 (red), as well as Dapi (blue) for nuclei. The images show 3D Z-stack confocal imaging (scale bar: *x*, *y*, and *z* = 100 μm) and their 2D orthogonal average intensity projections (scale bar: xy, xz and yz = 30 μm). **D** Quantification was done with ImageJ software by calculating the areas of positive ZO-1 staining and Dapi positive staining relative to the total area (*n* ≥ 9 samples per group, pooled from 3 independent experiments). **E** Enzyme-linked immunosorbent assay measurements show IFN-γ and IL-10 cytokine production in the basolateral side of the monolayer (*n* ≥ 6 samples per group, pooled from 2 independent experiments). Graphs show the mean ± SEM, * adjusted *p* ≤ 0.05, ** adjusted *p* ≤ 0.01, *** adjusted *p* ≤ 0.001. Significant differences were observed by using one-way ANOVA corrected with Dunnett´s multiple comparison test.

Innate immune cells, in particular macrophages, are key players in gut homeostasis, through direct killing of pathogens and thus preserving barrier function. Besides, they contribute to maintaining a balanced immune system by secreting cytokines and interacting with other types of immune cells^37^. Thus, we wanted to further investigate the role of macrophages as key players in homeostasis of the epithelial layer. Bone marrow progenitor cells were isolated from the femur and tibia of wild-type mice, cultured, and then polarized into M0, pro-inflammatory M1, and anti-inflammatory M2 subsets. Subsequently, they were co-cultured with monolayers for 24 h (Fig. 5A). Interestingly, M0 and anti-inflammatory M2 macrophage subsets had no significant effect on barrier integrity, as evidenced by TEER measurements and ZO-1 staining, which were comparable to monolayers cultured alone (Fig. 5B–D). However, co-cultures with M1 macrophages had the lowest TEER (147 ± 24.49%) compared to monolayers alone (215.6 ± 25.32%), but there was no significant effect on ZO-1. Furthermore, the cytokine production of macrophages after co-culture with monolayers was evaluated. As expected, M1 macrophages produced high IFN-γ (722.9 ± 67.86 pg/ml), while elevated IL-10 expression was observed in M2 macrophages (468.660 ± 67.69 ng/mL), relative to baseline levels in monolayers (Fig. 5E). This further emphasizes the dual role of macrophages in the gut as they are both guardians of intestinal homeostasis and drivers of inflammation^37,38^. Taken together, our findings show the successful establishment of functional co-culture models with macrophages, which demonstrated the effect of these immune cells on barrier integrity and cytokine production.

### Immune cell regulation of the epithelial barrier in response to pathogenic bacteria

Bacteria play an important role in the development and progression of IBD by affecting the integrity of the epithelial barrier and influencing the immune system. In healthy individuals, the gut microbiota helps maintain immune function and supports normal processes in the gut, while in IBD, the balance is disrupted, leading to an increase in harmful bacteria. This results in a dysregulated immune response, causing inflammation of the intestinal epithelium and thus disease development^39–41^. Therefore, to better understand the epithelial damage during bacterial infection, we first explored the effect of *Citrobacter rodentium*, an extracellular bacterium able to mimic microbial-driven mechanisms similar to IBD, to investigate its translocation dynamics^42,43^.

*C. rodentium* was added to the apical side of the monolayer culture and was left to incubate for a duration of 3, 6, and 24 h (Supplementary Fig. 5A). Bacterial infection resulted in a significant decrease in TEER, due to *C. rodentium's* ability to adhere to the epithelium as early as 3 h of incubation (Supplementary Fig. 5B–D). By hour 6, the bacteria translocated to the basolateral side, inducing cell death of epithelial cells (Supplementary Fig. 5E). We also tested the response of the monolayer to a more complex situation, where we first exposed it to pro-inflammatory factors like TNF-α and IFN-γ before bacterial infection (Supplementary Fig. 6A). Interestingly, IFN-γ alone or in combination with TNF-α led to a significant increase in barrier disruption and epithelial cell death, compared to untreated and TNF-α-treated monolayers, when exposed to *C. rodentium* (Supplementary Fig. 6B–D).

**Fig. 6:**
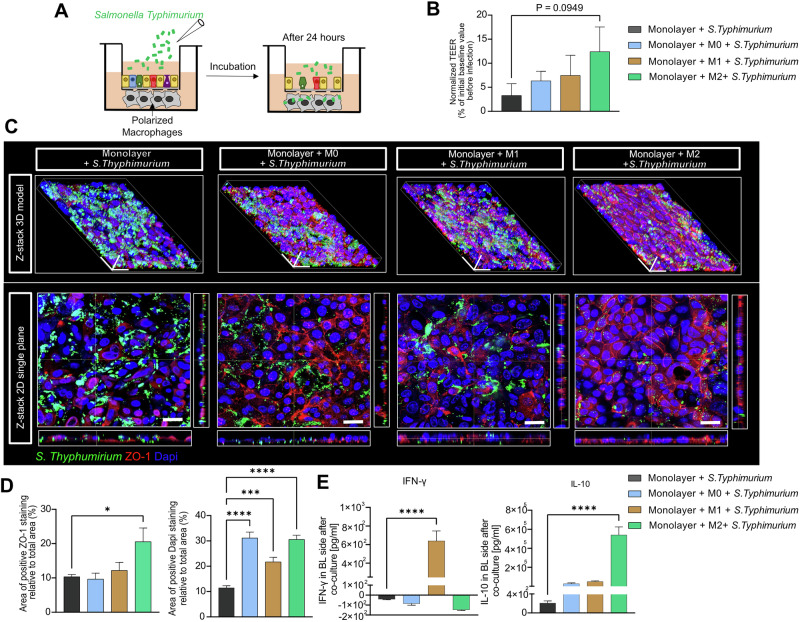
Protective role of macrophages against *Salmonella Typhimurium* infection. **A** Schematic representation of the experimental setup employed: co-culture of polarized macrophages and fully differentiated colon monolayer was set as previously described. After that, the co-culture model was infected with *S. Typhimurium* for 24 h. **B** Barrier integrity was assessed by TEER measurements (*n* ≥ 12 samples per group, pooled from 3 independent experiments). **C** Immunohistological stainings for tight junction protein ZO-1 (red), as well as Dapi (blue) for nuclei. The images show 3D Z-stack confocal imaging (scale bar: *x*, *y*, and *z* = 100 μm) and 2D single-plane projections (scale bar: xy, xz and yz = 30 μm). **D** Quantification was done with ImageJ software by calculating the areas of positive ZO-1 staining and Dapi positive staining relative to the total area (*n* ≥ 6 samples per group, pooled from 3 independent experiments). **E** Enzyme-linked immunosorbent assay measurements show IFN-γ and IL-10 cytokine production in the basolateral side of the monolayer (*n* = 6 samples per group, pooled from 2 independent experiments). Graphs show the mean ± SEM, * adjusted *p* ≤ 0.05, ** adjusted *p* ≤ 0.01, *** adjusted *p* ≤ 0.001. Significant differences were observed by using one-way ANOVA corrected with Dunnett´s multiple comparison test.

We have also tested the monolayer against intracellular bacteria, *Salmonella Typhimurium*, which caused strong disruption of the monolayer (Supplementary Movie 2). Compared to *C. rodentium*, *S. Typhimurium* was able to damage the epithelial layer as early as 15 min post-infection (data not shown). Complete disruption was evident after 3 h (data not shown). To understand the role of *S. Typhimurium* in more complex systems, we also co-cultured them with macrophages, as they are key players in defense against harmful pathogens. This triple co-culture system is shown in Fig. 6A. Monolayer co-culture with M2 macrophages led to increased barrier strength, as shown by TEER measurements (12.42 ± 3.91% compared to 3.28 ± 3.91% in monolayers with bacteria alone), whereas M0 and M1 macrophages had no significant effect on TEER values (Fig. 6B). Moreover, monolayer co-culture with M2 macrophages exhibited the highest ZO-1 area staining compared to M0 and M1, further highlighting their protective effect against *S. Typhimurium* infection (Fig. 6C, D). Interestingly, all macrophage subsets led to enhanced cell viability, as shown by increased DAPI staining compared to monolayers infected with bacteria alone. Additionally, M1 macrophages produced high levels of pro-inflammatory cytokine IFN-γ (639.3 ± 77.02 pg/ml), while M2 macrophages secreted elevated amounts of the anti-inflammatory cytokine IL-10 (540.163 ± 46.06 ng/ml) relative to baseline levels in monolayers with bacteria alone, showcasing their roles in promoting and regulating inflammation. These results demonstrate that our monolayer co-culture system is a valuable tool for studying the role of immune cells, particularly macrophages, in the immune defense against bacterial infections during IBD.

## Discussion

Inflammatory bowel diseases represent chronic disorders that can be triggered by disturbances in epithelial barrier integrity. The importance of the gut epithelium extends beyond that of a physical barrier; rather, it plays a crucial role by responding to diverse stimuli, including signals from the gut microbiota and the immune system^44^. In the pursuit of comprehending gut physiology, researchers have extensively used in vitro models, especially organoids. These 3D models not only serve as foundational tools for biological and medical investigations but also offer a means to replicate the aspects of the tissue morphology^16^. While organoids are valuable in gut research, their enclosed structures prevent access to the epithelium, which limits their applications, making it challenging to study barrier function^21^.

Monolayer systems, on the other hand, are particularly advantageous for a variety of reasons, one of which is that they provide direct access to the epithelium. In vitro monolayer models were often based on conventional colorectal cancer cell lines such as the Caco-2 cell line, which could be used for modeling the intestinal epithelium and understanding barrier integrity. While monolayer models based on cancer cell lines help with the overall understanding of the gut physiology, they are not always suitable for translation to more complex conditions in the gut, as they do not replicate the behavior of primary intestinal epithelial cells^45^. In IBD, particularly, a gap remains in the development of a readily reproducible in vitro colonic system for studying intestinal epithelial changes during inflammatory settings while also incorporating the immune system. In this study, we report the development of a novel murine epithelial colon monolayer model that is ready to be used in a variety of IBD-like conditions. One of the main features of our model is the incorporation of immune cell compartments consisting of polarized T cells and macrophages, both known for their important roles in IBD, making its features close to the diverse and functional state of the in vivo status^6^.

The proposed model was developed by directly isolating colon crypts, digesting them, and subsequently placing the single cells onto Matrigel-coated transwells for cultivation. Following a 7-day cultivation period, we successfully achieved a fully confluent monolayer, demonstrating the characteristics expected from the in vivo colon. Existing studies showed that colon monolayers can also be grown from previously cultured organoids^46,47^. However, as we have demonstrated, such protocols are time-consuming, requiring large amounts of materials and approximately 21 days to achieve confluent monolayers. While a single study reported the successful development of colon epithelial monolayers directly from mouse tissue, no functional evaluation similar to in vivo-like conditions has been reported^48^. Unlike the models using the Caco-2 cell line or the approach reported by Kozuka et al., our model not only simplifies the process of monolayer growth but also results in a fully grown epithelial layer with in vivo-like properties within a matter of several days. We were also able to maintain the monolayer in culture for up to 22 days by changing the medium daily. This is particularly advantageous, since it could allow long-term monitoring of intestinal epithelial behaviors.

To characterize our model on a cellular level, we showed that the system exhibited the expression of in vivo colon epithelial markers, mainly enterocytes expressed by Chga, responsible for metabolic processes, goblet cells marked by Muc2, which are responsible for mucin production, and Lgr5+ stem cells^49^. One of the key features of colon epithelium is the formation of tight junctions, which are crucial for preserving the integrity of the intestinal barrier and regulating the paracellular movement of various substances, including ions and solutes, across the intestinal epithelium^50^. In our study, we specifically examined the expression of tight junction markers, and we therefore reported the expression of ZO-1, Claudin-1, Claudin-2, and Occludin (data not shown), aligning with previous findings that explore the in vivo physiological characteristics of the colon^50^.

Furthermore, the functionality of this model was evaluated based on its capability to mimic characteristics similar to those observed in IBD, mainly including cell death, barrier integrity and bacterial translocation. In the context of IBD, cytokines have been considered as key signaling molecules in the intestinal immune system, exerting a direct influence on the pathogenesis of the disease while also regulating gut inflammation^1,51^. Specifically, TNF-α and IFN-γ, which are key proinflammatory mediators, are significantly increased in IBD, leading to intestinal epithelial cell death and disruption of the gut epithelial barrier function^29,52,53^. IFN-γ has been shown to drive IBD pathogenesis through immune-modulatory effects, while TNF-α is increased in the mucosa and intestinal wall of patients with IBD, and its overactivity is a crucial mediator of the abnormal immune response^1,28^. Thus, we stimulated the monolayers with TNF-α, IFN-γ or a combination of both to study their effect on barrier integrity. We report that IFN-γ treatment, or in combination with TNF-α, induced higher cell death and increased permeability. This can be attributed to its ability to affect the expression of tight junction proteins, including ZO-1, which are essential for maintaining the integrity of the intestinal barrier^54,55^. Regarding TNF-α, different studies have reported its effects in modulating epithelial barrier function, including tight junction organization, as well as stem cell activity^56–58^. Particularly, in epithelial cells, TNF-α can promote either survival or cell death, depending on the downstream signaling. For instance, when TNF-α signals through TNFR1 or TNFR2, it can lead to activation of nuclear factor-κB (NF-κB), a pathway that generally promotes cell survival. In contrast, TNF-α signaling through non-ubiquitylated RIPK1 can trigger cell death processes, including apoptosis or necroptosis^59^. This observation is consistent with other studies, which have shown that TNF-α signaling can contribute to tissue regeneration and repair rather than tissue damage. In this context, TNF-α functions as a regeneration-inducing cytokine that promote epithelial proliferation and barrier restoration^60,61^. In addition, it has been reported that TNF-α alone might have a minimal effect on epithelial cells unless it is combined with IFN-γ. This effect can be explained by the upregulation of TNFR2 by IFN-γ, which enables TNF-α to more efficiently engage its receptor, therefore leading to epithelial barrier dysfunction and cell death^57,62^. This mechanism is consistent with our observations, as significant effects on the monolayers were only detected when TNF-α was combined with IFN-γ. Treatment with TNF-α alone did not induce cell death, therefore suggesting activation of signaling pathways that promote cell survival rather than cell death. Taken together, our data show that the presented model can be used to study the functional effect of a variety of mediators, such as pro-inflammatory cytokines, on epithelial barrier integrity.

Another important aspect in the pathogenesis of IBD is epithelial-immune cell interactions. For example, innate immune cells such as macrophages and dendritic cells are key antigen-presenting cells found in the inflamed mucosa and are known to produce large amounts of inflammatory cytokines. Also, adaptive immune cells, including effector T cells, have been highly detected in inflamed bowel and were associated with the secretion of pro-inflammatory cytokines^1^.

Thus, to better mimic the in vivo condition, we were interested in incorporating an immune cell compartment into the already developed epithelial monolayer model. Interestingly, polarized Th1 and Th17 cells lead to an increase in epithelial permeability along with a decrease in tight junction protein ZO-1. This was also associated with high production of pro-inflammatory cytokines IFN-γ and IL17 in co-cultures with Th1 and Th17, respectively. Notably, a previous study by Camoglio et al. reported that IFN-γ production by lamina propria is increased in patients with Crohn’s disease compared with patients with ulcerative colitis and healthy controls^63^. Similarly, Fuss et al. showed that lamina propria T cells from inflamed Crohn’s disease mucosa have increased IFN-γ secretion compared with control LP T cells, while LP T cell production of IFN-γ in ulcerative colitis remains normal^64^. Also, it has been shown that there is increased production of Th17 cell-associated cytokines, such as IL-17a and IL-17F, by lamina propria T cells in both Crohn’s disease and ulcerative colitis, which aligns with our observations^1^. Next, we developed co-culture models of macrophages with epithelial monolayers. Interestingly, we could show that under steady state conditions, M0, M1, and M2 macrophages had no significant effect on barrier integrity. This suggests that in the absence of pro-inflammatory stimuli, macrophages are maintaining the homeostasis of the epithelial barrier, which could potentially help preserve its integrity^38^. To further explore the role of macrophages in response to external stimuli, we decided to infect the macrophage co-cultures with *Salmonella Typhimurium*.

It is known that pathogenic and commensal bacteria have been a subject of extensive research in IBD. Specifically, the onset and development of IBD could be attributed to aberrant immune responses directed toward alterations in gut microbiota or, in some cases, bacterial infections^65,66^. In this context, we show that *S. Typhimurium* infection caused significant disruption to the epithelial layer, as evidenced by a decrease in TEER and ZO-1 expression. Interestingly, our findings suggest that M2 macrophages played a beneficial role by producing the anti-inflammatory cytokine IL-10. This production of IL-10 may have contributed to the restoration of TEER and the increased expression of ZO-1, compared to monolayers cultured alone. On the other hand, M1 macrophages produced IFN-γ, which could also lead to damage in the epithelial layer, as evidenced by a decrease in TEER. Taken together, these results prove the important role of macrophages during bacterial infection in the gut, and to our knowledge, we are the first to establish a triple co-culture mouse monolayer model that includes polarized macrophages and uses *S. Typhimurium* as an infection model in vitro. We have also used an extracellular bacterium in order to study bacterial translocation through the monolayer in a time-dependent manner, using the *C. rodentium* infection model. In murine studies, *C. rodentium* infection has been shown to induce disruptions in the colonic epithelial barrier and lead to colonic mucosal thickening, which is similar to that observed in IBD^67^. Similarly, we observed a significant increase in cell death rates and permeability following bacterial infection, particularly evident after 24 h. Most importantly, our findings illustrated that the mechanism of action of *C. rodentium* depends on its translocation. The bacteria became adherent to the epithelial layer after only 3 h of incubation and subsequently translocated from the apical side to the basolateral side, similar to what is observed in in vivo situations^68^. Also, we demonstrated that *C. rodentium* exhibited accelerated translocation through the colon monolayer model when pre-treated with IFN-γ and TNF-α. This acceleration was concomitant with increased cell death and a decrease in tight junction features. These findings emphasize the significant role of TNF-α and IFN-γ in the dynamics of bacterial translocation in IBD. In line with previous findings, this disruption resulted in increased permeability and excessive bacterial translocation^28,69,70^.

In summary, this study outlines the establishment of a murine colon epithelial monolayer model that allows the investigation of key features of gut inflammation and modeling of IBD dynamics, including epithelial barrier function, immune cell interactions, and bacterial translocation. The monolayer model represents in vivo-like epithelial lineages, including strong barrier function and integrity. By incorporating an immune cell compartment of polarized T cells and macrophages, we demonstrated the impact of activated immune cells on epithelial integrity, with pro-inflammatory Th1/Th17 cells promoting barrier damage while anti-inflammatory M2 macrophages contributing to tissue repair. Moreover, the model was used to study bacterial infection, including *Salmonella Typhimurium*, which compromised barrier function. In response, macrophages played a protective role by reducing epithelial damage and reinforcing tight junction protein expression. Although we demonstrated the diverse applications of the current model, it still has some limitations. For instance, we only reported the development of the monolayer from healthy tissue and did not address inflamed or diseased models (data not shown). The model also lacks other cellular compartments, such as fibroblasts and endothelial cells. Future work will focus on incorporating more cell types in order to address the limitations of the current model. Besides, we are working on adapting the model to a human setting, which will enable us to investigate these interactions in a more clinical and patient-focused manner. We strongly believe that the application of this model will significantly enhance our comprehension of the mechanisms regulating barrier integrity in gut inflammation.

## Methods

### Colon crypt isolation and epithelial monolayer culture

All experiments were performed according to the State Government of Middle Franconia Institutional Animal Care and Use Committee. In this study, C57BL/6J mice were bred in-house and kept in individually ventilated cages under specific-pathogen-free conditions at the Department of Medicine 1, Universitätsklinikum Erlangen. Male and female C57BL/6J mice, aged 8–12 weeks, were anesthetized using a 1.5–2% isoflurane-oxygen mixture in an approved chamber. Once deep anesthesia was confirmed, animals were euthanized by cervical dislocation, and the colon was immediately isolated (Supplementary Figure 1). Afterward, it was flushed with cold phosphate buffered saline (PBS), opened longitudinally and cut into 3–4 mm pieces. Colon pieces were washed repeatedly with cold PBS to remove any remaining stool. After PBS removal, crypt isolation buffer (CIB: 30 ml PBS + 2 mM ethylenediaminetetraacetic acid (EDTA) was used to digest the tissue for 1 h at 4 °C on a rotator (RS-TR05, Phoenix Instruments). CIB was then replaced with cold PBS, and colon pieces were shaken vigorously for at least 2 min. The crypt fraction was collected and kept on ice. This step was repeated at least 5 times to release the majority of crypts from the tissue. The crypt fraction was centrifuged for 5 min at 1400 rpm at 4 °C, and the pellet was washed twice with washing buffer (WB: DMEM (Gibco) + 0.04% Bovine Serum Albumin (BSA, Carl Roth)). To obtain a single cell suspension, 2 ml of pre-warmed Triple E Express (Gibco) was added to the pelleted crypts and incubated for 20 min at 37 °C. During this time, the pelleted crypts were vortexed every 3–4 min, to promote the cell dissociation. To stop the reaction, WB was added, and the cells were strained through a 100 μm cell strainer. They were then centrifuged at 1400 rpm for 5 min, counted and resuspended in media, also referred to as crypt culture media (CCM). CCM consisted of Advanced DMEM/F-12 (Gibco), 1% Penicillin/Streptomycin (P/S, Sigma-Aldrich), 10 mM 4-(2-hydroxyethyl)-1-piperazineethanesulfonic acid (HEPES), 1% GlutaMAX (Gibco), 1X B-27 supplement (Gibco), 1mM N-Acetyl-L-cysteine (NAC, Sigma Aldrich), 50 ng/ml recombinant murine Epidermal Growth Factor (rmEGF, ImmunoTools), 100 ng/ml recombinant murine Noggin (rmNoggin, PeproTech), 0.05 nM Wnt3a (Uprotein express) and (10% R-Spondin (developed in house from R-Spondin cell line). To ensure stem cell survival, 10 µM dihydrochloride (y-27632, AdcoQ) was also added in the first 2–3 days of culture. Transwell cell culture inserts (6.5 mm, 0.4 μm or 3 μm pore size, Cellqart) were coated with Matrigel (1:10 in PBS, Cultrex, R&D Systems) and incubated at 37 °C for 1 h. After polymerization, PBS was gently removed, and 10^5^ cells/well were added to the apical compartment. CCM was also added to the basolateral compartment of 24-well plates. On day 3 of culture, pre-warmed differentiation media consisting of CCM without y-27632 was added to both the apical and basolateral compartments and the monolayers were incubated at 37 °C until full confluence was reached (typically after 7 days in culture).

### Cytokine stimulation

To study the effect of pro-inflammatory cytokines on barrier integrity, colon monolayers were stimulated on the apical and basolateral sides with 1 μg/ml of Tumor Necrosis Factor alpha (TNF-α, Biolegend) and Interferon-gamma (IFN-γ, Biolegend) for 24 h. Transepithelial electrical resistance (TEER) was then measured, and permeability assays were performed. Monolayers were also collected for immunofluorescence staining as described below.

### Establishment of a co-culture system of epithelial monolayer and polarized immune T cells

The spleens of wild-type C57BL/6 mice were harvested and then gently smashed between two glass slides. The cell suspension was collected in RPMI 1640 medium (Sigma-Aldrich) supplemented with 10% Fetal Bovine Serum (FBS, PAN-Biotech) and 1% P/S (Sigma-Aldrich) and centrifuged at 1400 rpm for 5 min. The supernatant was discarded, and ACK lysis buffer (4.45 g NH₄Cl + 0.5 g KHCO₃ + 18.6 mg EDTA in 500 ml Millipore water, pH 7.2) was added to the pellet to lyse erythrocytes, followed by incubation for 3 min at room temperature. The reaction was stopped by adding 20 ml of complete RPMI medium, and the suspension was filtered through a 40 µm cell strainer. After counting, CD4+ T cells were isolated using the CD4+ isolation kit (Miltenyi Biotec), followed by isolation of CD25- cells using the CD25- isolation kit (Miltenyi Biotec), according to the manufacturer’s instructions. Both work kits work based on negative selection by labeling all cells that are not CD4+ or not CD25- with magnetic beads. The labeled cells are retained in a magnetic column, while the unlabeled cells, which are CD4+CD25-, pass through and are collected.

0.5 × 10^6^ cells were seeded in pre-coated 24-well plates with anti-CD3 (10 µg/ml, BioXCell) and anti-CD28 (10 µg/ml, BioXCell). The cells were cultured in RPMI 1640 medium (Sigma-Aldrich) supplemented with 10% Fetal Bovine Serum (FBS, PAN-Biotech) and 1% P/S (Sigma-Aldrich). For Th1 polarization, the cells were stimulated with interleukin (IL)-12 (20 ng/ml, R&D Systems) and anti-IL4 (5 µg/ml, BioXCell). For Th17 polarization, the cells were supplemented with anti-IL4 (5 µg/ml, BioXCell), anti-IFNγ (5 µg/ml, produced in-house), IL-6 (100 ng/ml, Miltenyi Biotech), IL-23 (50 ng/ml, Miltenyi Biotech), and IL-1β (20 ng/ml, ImmunoTools). The cells were kept in culture for 3 days at 37 °C. Unpolarized cells, considered Th0, served as a control. Finally, the medium was changed into complete CCM, and the monolayers were put in co-culture with T cells for 24 h at 37 °C, followed by preparation for further analysis.

### Establishment of a co-culture system of epithelial monolayer and polarized macrophages

Macrophages were differentiated from murine bone marrow progenitor cells of C57BL/6 mice, as previously described^71^. Briefly, femurs and tibias were harvested and flushed with PBS to collect the cells, which were resuspended in DMEM/F12 (Anprotec) supplemented with 10% FBS (PAN-Biotech) and 1% P/S (Sigma-Aldrich). To promote macrophage differentiation, a total of 0.5 × 10^6^ cells were plated in 24-well plates and cultured in the presence of 20 ng/ml of Macrophage Colony-Stimulating Factor (M-CSF, Miltenyi Biotech) for 7 days at 37 °C. On day 3, an additional 500 µL of supplemented DMEM/F12 containing M-CSF was added to the cultures. On day 7, macrophage polarization was induced by resuspending the cells in 1 mL of RPMI (Gibco) supplemented with 10% FBS (PAN-Biotech) and 1% P/S (Sigma-Aldrich). For M1 polarization, cells were stimulated with 100 ng/ml of Lipopolysaccharide (LPS, Sigma-Aldrich) and 50 ng/ml of IFN-γ (Biolegend), while for M2 polarization, cells were treated with 20 ng/ml of IL-4 (ImmunoTools). Unstimulated cells, considered M0, served as a control. The macrophages were incubated for an additional 2 days at 37 °C before proceeding to co-culture experiments. The medium was changed into complete CCM, and the monolayers were put in co-culture with macrophages for 24 h at 37 °C, followed by preparation for further analysis.

### *Salmonella Typhimurium* infection and experimental setup of the triple co-culture model with polarized macrophages

To study the role of macrophages in response to bacterial infection of the intestinal epithelium in the context of IBD, we used an Ampicillin-resistant and GFP fluorescent strain of *Salmonella Typhimurium* (*S. Typhimurium*). *S. Typhimurium* was grown in sterile LB medium (Roth) supplemented with Ampicillin (100 μg/ml, Inresa Arzneimittel GmbH) at 37 °C under constant shaking conditions. Optical density at 600 nm (OD600) was measured to assess bacterial growth. When *S. Typhimurium* reached exponential growth (OD600 = 1), it was centrifuged at 5000 × *g* for 10 min and resuspended in CCM without P/S. Colon monolayers were put in co-culture with macrophages (M0, M1, and M2) and were then infected by adding bacterial fraction to the apical side and incubated at 37 °C. After 24 h, the monolayers were carefully washed with warm PBS, and TEER was measured. Monolayers were fixed for staining, and basolateral media were also collected for cytokine measurements.

### *Citrobacter rodentium* infection and translocation model

To study another type of infection and translocation in colon monolayers, we used an erythromycin-resistant and mCherry fluorescent strain of *Citrobacter rodentium* (*C. rodentium*; strain ICC169). *C. rodentium* was grown in sterile LB medium (Roth) supplemented with erythromycin (500 μg/ml, Inresa Arzneimittel GmbH) at 37 °C under constant shaking conditions. When *C. rodentium* reached exponential growth (OD600 = 1), it was centrifuged at 5000 × *g* for 10 min and resuspended in CCM without P/S. Colon monolayers were then infected by adding *C. rodentium* to the apical side and incubated at 37 °C. After 3, 6, and 24 h, the monolayers were carefully washed with warm PBS, and TEER was measured. Monolayers were fixed for staining. Uninfected monolayers were used as controls for each experiment. Basolateral media were also collected, and the translocated bacteria were imaged using a confocal microscope (TCS SP5, Leica) and quantified using ImageJ software (National Institutes of Health).

### Transepithelial electrical resistance measurement (TEER) and normalization

To assess barrier development and its integrity, TEER was measured using a world precision instrument, the EVOM3 Epithelial Voltmeter (Millicell® ERS), according to the manufacturer’s instructions. Briefly, STX4 electrodes were sterilized in 70% ethanol for 10 min and then shortly immersed in PBS. Measurements were taken immediately after adding fresh media to the transwells. TEER values were calculated as following:

$$TEER\left(Ohm\,x\,c{m}^{2}\right)=\left(Rs-Rb\right)\,{\rm{x}}\,A$$

where Rs is the resistance of the insert with the monolayer (in Ohms) at a given time, Rb is the resistance of an empty insert without cells (in Ohms), and A is the surface area of the insert (in cm^2^). To account for variability in TEER values across wells and experiments, TEER normalization was performed as previously described by Garcia-Morena et al.^72^. For this, TEER values measured for each fully formed monolayer immediately before co-culture or infection was defined as the baseline and set to 100%. After the co-culture or infection was initiated, TEER was measured again at the indicated time points, and values are expressed as percentages relative to this initial baseline:

$$Normalized\,TEER\,\left( \% \right)=\frac{\mathrm{TEER}\,\mathrm{after}\,\mathrm{coculture}\,\left(\mathrm{Ohm}\,x\,{\mathrm{cm}}^{2}\right)}{\mathrm{TEE}\,\mathrm{basline}\,\mathrm{before}\,\mathrm{coculture}\,\left(\mathrm{Ohm}\,x\,{\mathrm{cm}}^{2}\right)}{\rm{x}}100$$

### Fluorescein permeability assays

The permeability of the colonic epithelial monolayer model was assessed using a fluorescein permeability assay. For this, fluorescein (5 mg/ml, Sigma) was reconstituted in CCM. On the basolateral side, CCM medium without fluorescein was added. Transwells were incubated at 37 °C for 30 min up to 6 h. For permeability assessment, 100 μl of media from the basolateral side was transferred to a 96-well plate. The absorbance was measured at 460 nm using a TECAN NanoQuant Infinite M200 plate reader. Apparent permeability (Papp) values were calculated using the following equations:

$$Papp=\frac{dQ/dt}{C0\,{\rm{x}}\,A}$$

defined as:

Papp = Apparent permeability coefficient (cm/s).

d*Q*/d*t* = Rate of fluorescein permeability (mg/s).

*A* = Surface area of the monolayer (cm^2^).

*C*_0_ = Initial fluorescein concentration in the apical side (mg/mL).

### Immunofluorescence staining

Immunofluorescence staining was used to evaluate the expression of various epithelial, tight junction and cell death markers (Supplementary Figure 2). Monolayers were fixed with 4% paraformaldehyde (PFA), permeabilized with 0.5% Triton, washed with PBS and blocked with TBST buffer containing tris-buffered saline, 1% Tween 20 (Sigma-Aldrich) and 2% BSA (Carl Roth). The following primary antibodies were added, and the monolayers were incubated overnight at 4°C: MUC2 (Novus Biologicals), CHGA (Novus Biologicals), Lgr5/GPR49 (Novus Biologicals), EpCAM (Invitrogen), Ki67 (Abcam) and ZO-1 (Invitrogen). For visualization, anti-rabbit-AlexaFluor555 secondary antibody was added for 1 h at room temperature (RT). For cell death, Propidium Iodide (PI, ThermoFisher Scientific) or Sytox (Invitrogen) was used. Cell nuclei were counterstained with 40,6-diamidino-2-phenylindole (Dapi) for 20 min at RT. The monolayers were carefully cut and mounted on a glass slide using fluorescent mounting medium (Dako). For imaging, a confocal microscope (TCS SP5, Leica) was used. The data were analyzed using ImageJ software (National Institutes of Health).

### Enzyme-linked immunosorbent assay

IFN-γ, IL-17 and IL-10 ELISA kits (Biolegend) were used as described in the manufacturer’s protocols.

### Statistical analysis

Data were analyzed using GraphPad Prism 10 (GraphPad Software). For column analysis, one-way ANOVA with Dunnett’s correction was used. For group analysis, two-way ANOVA with Šídák’s multiple comparisons test was used. Significant changes were marked as follows: *p ≤ 0.05, **p ≤ 0.01, ***p ≤ 0.001 and ****p ≤ 0.0001.

## Supplementary information

Supplementary Information

Supplementary Movie1

Supplementary Movie2

## Data Availability

The data are available upon reasonable request to the corresponding authors.
