# Supplementary material for: In vitro modeling of inflammatory bowel diseases using a newly developed immunocompetent colon epithelial monolayer co-culture model
Source: NPJ Biomed Innov. 2026 Jun 15;3:37. doi: 10.1038/s44385-026-00091-9 (PMC13269889; doi:10.1038/s44385-026-00091-9)
Supplement: Supplementary file 1 — Supplementary Information [file 44385_2026_91_MOESM1_ESM.pdf]

Supplementary Information

Title

***In vitro* modeling of inflammatory bowel diseases using a newly developed immunocompetent colon epithelial monolayer co-culture model**

*Author(s), and Corresponding Author(s)**

Imen Larafa[*]*, Roxana Zogorean, Adrian Bühler, Claudia Günther, Stefan Wirtz, Markus F. Neurath, Oana-Maria Thoma, Maximilian J. Waldner*

Supplementary Figures

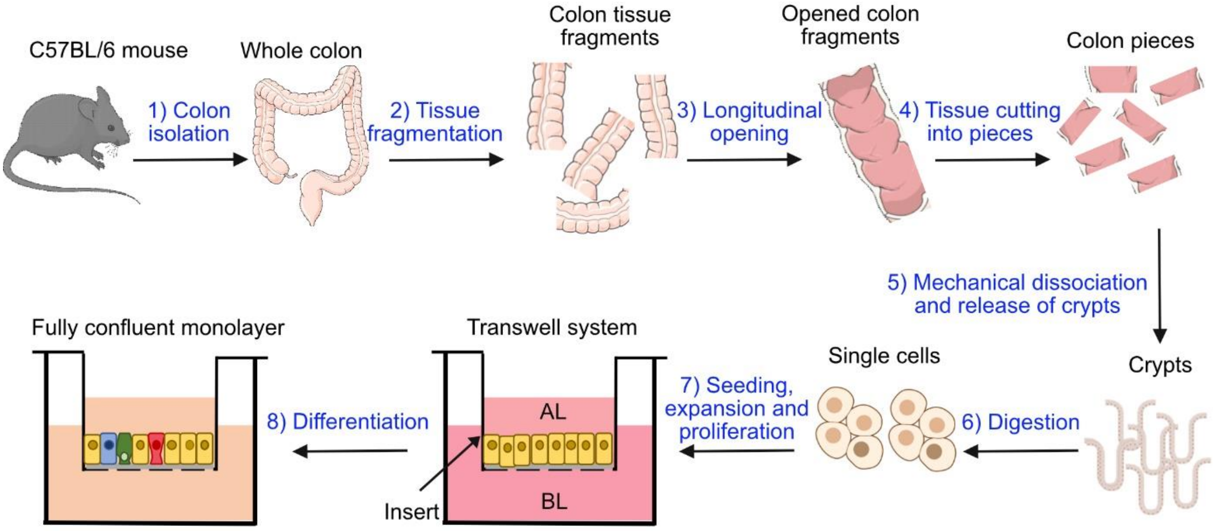

Supplementary Figure 1. Schematic representation of the experimental setup used to generate murine colon epithelial monolayer cultures. Colon tissue was isolated from wild-type C57BL/6 mice and fragmented into 4-5 pieces. The tissue was opened longitudinally, and each fragment was cut into 3–4 mm pieces followed by digestion in CIB buffer. CIB was subsequently replaced with PBS, and the tissue fragments were vigorously shaken to release the crypts. The isolated crypts were then digested into single cells and washed with wash buffer (WB). In parallel, transwell inserts were coated with a thin layer of Matrigel. The isolated epithelial cells were seeded onto the coated transwells in seeding medium for 3 days. Afterwards, the medium was replaced with differentiation medium, and cultures were maintained until fully confluent and differentiated. The image contains elements from SMART Servier Medical Art under Servier licensed Creative Commons Attribution 4.0 International.

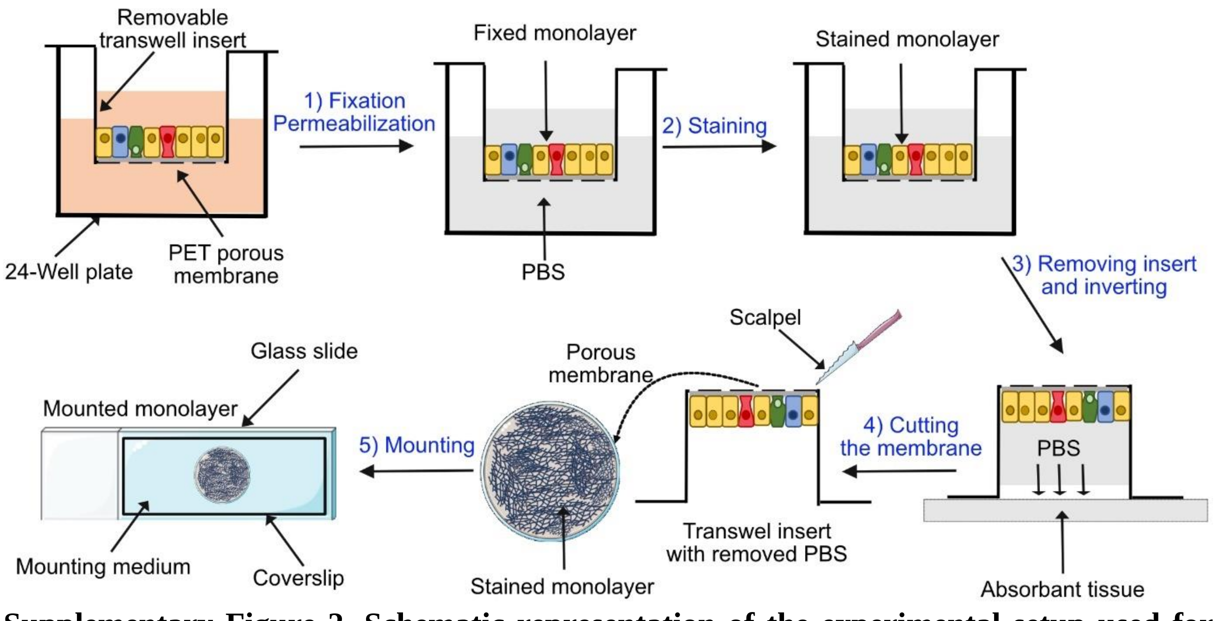

Supplementary Figure 2. Schematic representation of the experimental setup used for staining and mounting colon epithelial monolayer cultures. Once fully grown, the culture medium was removed and the monolayers were shortly washed with PBS. The cells were then fixed with 4% paraformaldehyde (PFA) and washed again with PBS. For permeabilization,

0.5% Triton X-100 was added and subsequently replaced with PBS. Immunostaining was performed using primary and secondary antibodies. After staining, the monolayers can be stored at 4 °C until mounting. For mounting, the inserts were inverted to remove excess PBS, and the PET membrane on which the monolayer was grown carefully cut out using a scalpel. It was then placed using a forceps onto a glass slide with the PET membrane facing the slide and the monolayer facing upward. Mounting medium was added on top of the monolayer, and the sample was covered with a coverslip. The mounted monolayers were incubated at 4 °C for 24 hours and imaged using confocal microscopy (TCS SP5, Leica). The image contains elements from SMART Servier Medical Art under Servier licensed Creative Commons Attribution 4.0 International.

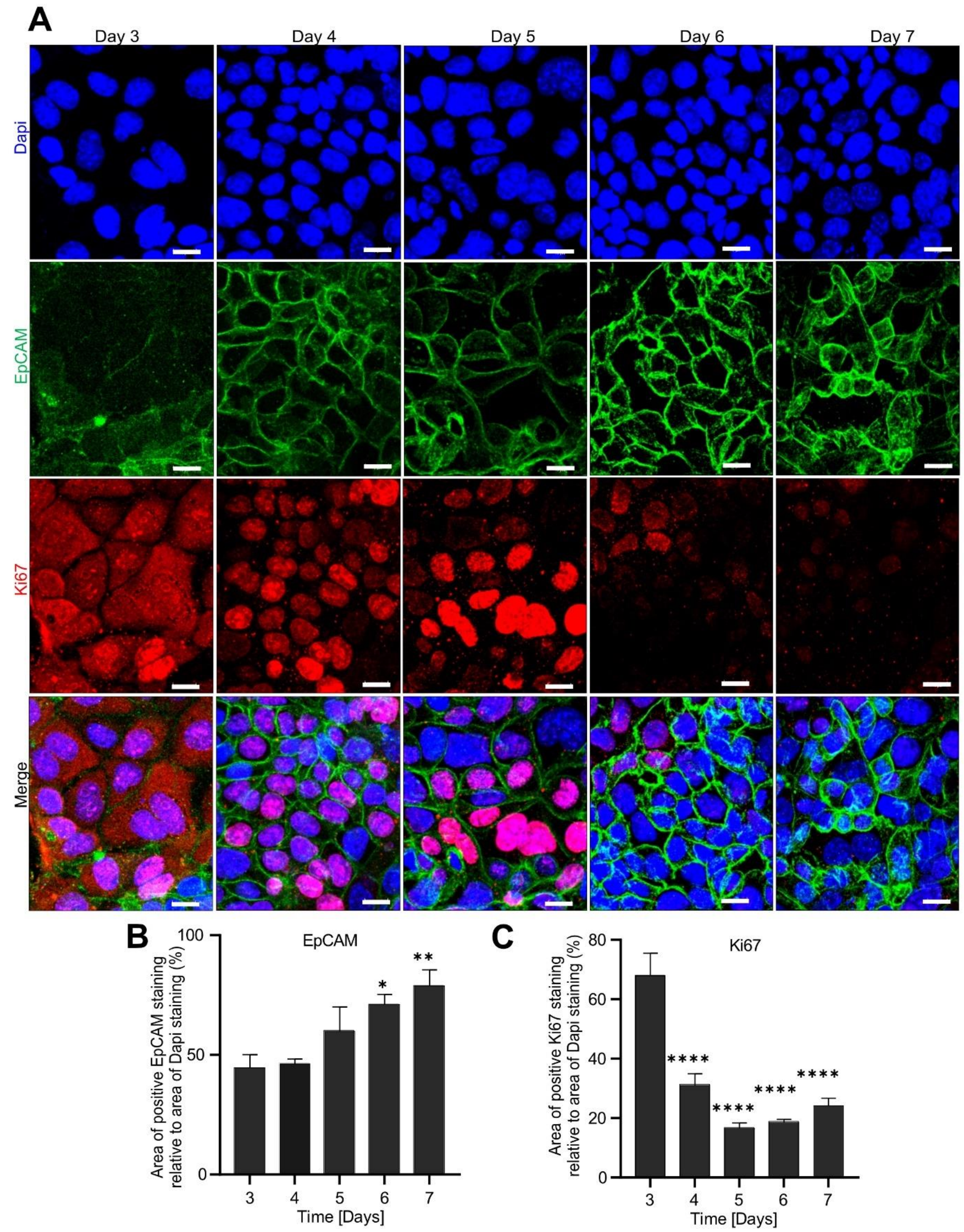

Supplementary Figure 3. Daily monitoring of epithelial and proliferation markers in the colon epithelial monolayer cultures. (A) Immunohistochemistry staining for EpCAM (green) and Ki67 (red) in daily collected monolayers from day 3 to day 7. Cell nuclei were counterstained with Dapi (scale bar = 10 µm). (B-C) Quantification was done by calculating the area of positive staining relative to the area of Dapi staining (n =4 samples per group pooled from 2 independent experiments). The data show the mean ± SEM, * adjusted $p \leq 0.05$, ** adjusted $p \leq 0.01$, *** adjusted $p \leq 0.001$. Statistical differences were calculated compared to day 3 using one-way ANOVA with Dunnett´s multiple comparisons test.

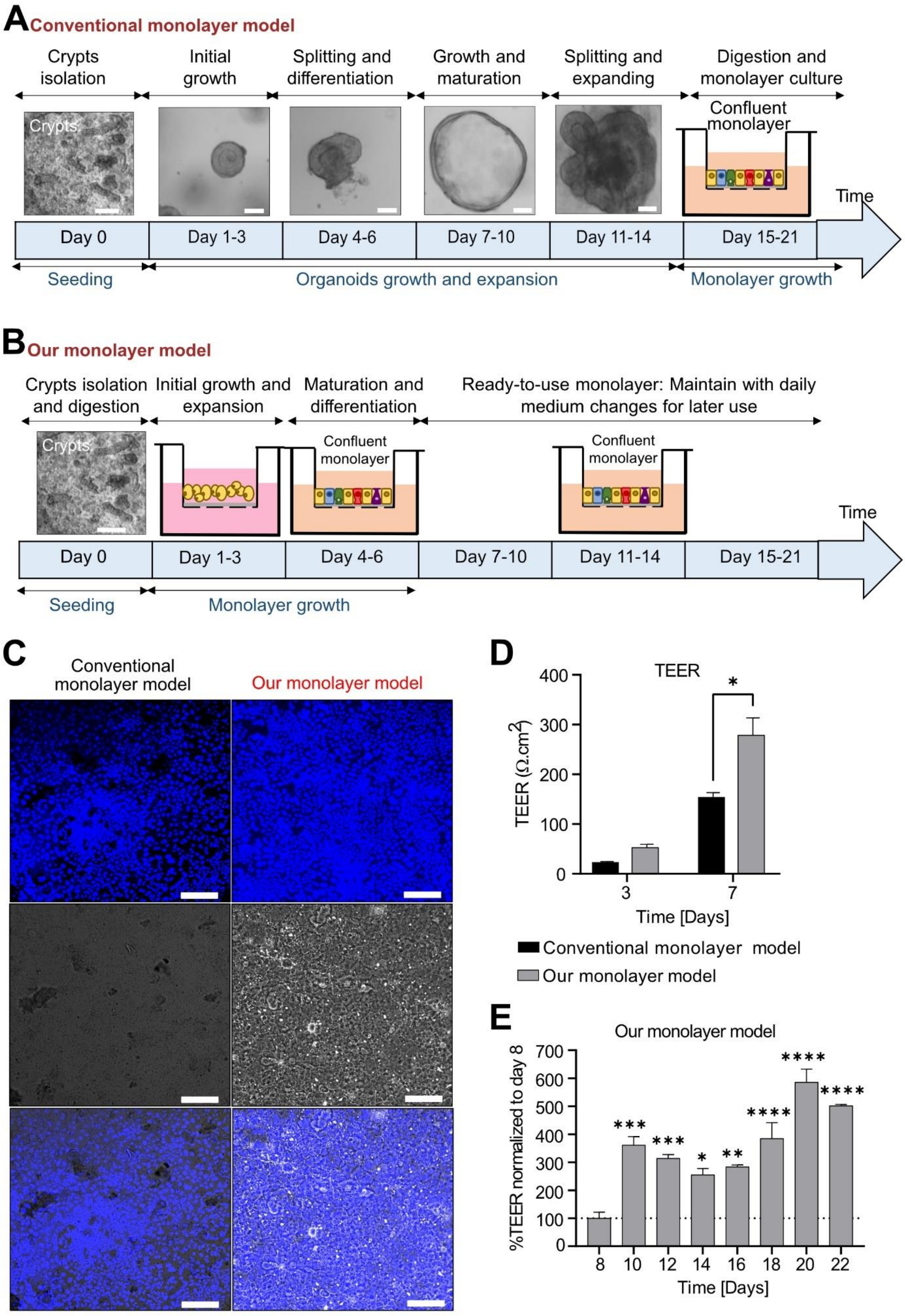
A
Conventional monolayer model
Crypts isolation
Initial growth
Splitting and differentiation
Growth and maturation
Splitting and expanding
Digestion and monolayer culture
Crypts
Confluent monolayer
Time
Day 0
Day 1-3
Day 4-6
Day 7-10
Day 11-14
Day 15-21
Seeding
Organoids growth and expansion
Monolayer growth
B
Our monolayer model
Crypts isolation and digestion
Initial growth and expansion
Maturation and differentiation
Ready-to-use monolayer: Maintain with daily medium changes for later use
Crypts
Confluent monolayer
Confluent monolayer
Time
Day 0
Day 1-3
Day 4-6
Day 7-10
Day 11-14
Day 15-21
Seeding
Monolayer growth
C
Conventional monolayer model
Our monolayer model
D
TEER
TEER (Ω.cm2)
400
300
200
100
0
*
3
7
Time [Days]
Conventional monolayer model
Our monolayer model
E
Our monolayer model
%TEER normalized to day 8
700
600
500
400
300
200
100
0
***
***
*
**
****
****
****
8
10
12
14
16
18
20
22
Time [Days]

Supplementary Figure 4. Comparison between our developed monolayer model and traditional monolayer system. (A) Schematic representation of the traditional monolayer model generated from colon organoids: Crypts were isolated from mouse colon and were plated into 3D Matrigel matrix for 3 days at 37°C with seeding medium. The seeding medium was changed into differentiation medium and the 3D organoids were kept in culture until they start growing (6 days). The organoids were then split twice per week and maintained in culture until fully grown (14 days). They were then collected and digested into single cells and then plated into Matrigel coated transwell system until fully grown (7 days). (B) Our model was generated directly from digested crypts as previously described, and is ready to use after only 7 days. (C) Immunohistochemistry staining was performed on monolayers from the traditional protocol (7 days after plating the digested organoids) compared to our newly developed system at day 7. Nuclei were stained with Dapi (blue), scale bar = 100 µm. (D) TEER measurements were performed at day 3 and day 7 after plating the single cells from both methods ($n \geq 6$ samples per group pooled from 2 independent experiments). (E) Long term monitoring of TEER ($n = 3$ samples per group except for day 20-22 where only $n = 2$ samples per group were obtained, pooled from 2 independent experiments). Graphs show the mean ± SEM, * adjusted $p \leq 0.05$, ** adjusted $p \leq 0.01$, *** adjusted $p \leq 0.001$. Statistical differences were calculated using one-way ANOVA corrected with Dunnett´s multiple comparisons test and with two-way ANOVA with Šídák’s multiple comparisons test for grouped analysis.

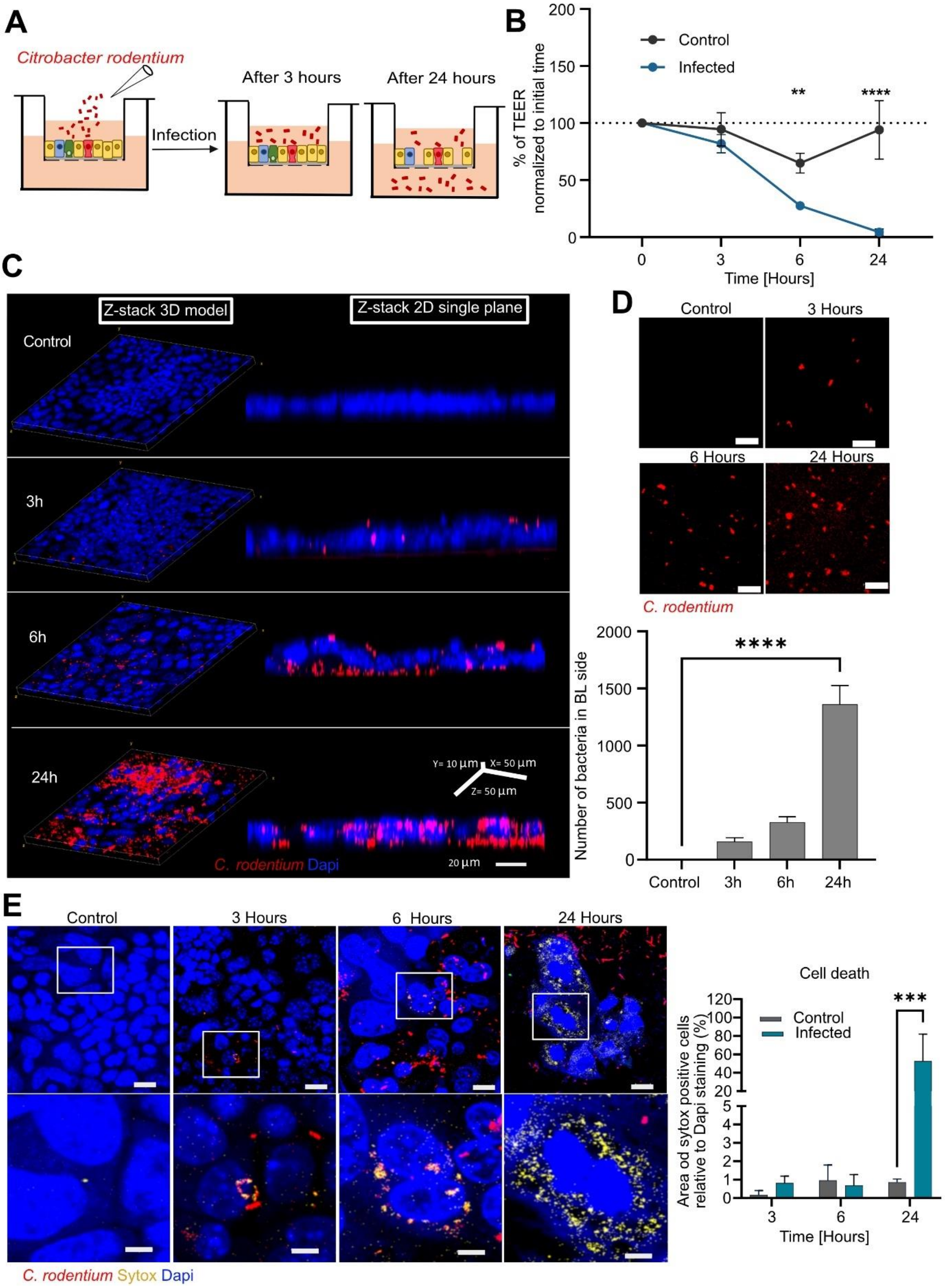

Supplementary Figure 5. *Citrobacter rodentium* translocates across the monolayer while inducing barrier disruption and cell death. (A) Colon monolayers were infected with *C. rodentium* for 3h, 6h and 24h. (B) Barrier integrity was assessed by TEER measurements (n ≥ 7 samples per group, pooled from 3 independent experiments). (C) Bacteria translocation

through the monolayer was investigated by immunohistochemistry staining followed by Z-stack confocal imaging. (D) The number of bacteria in the basolateral side (BL) was quantified by ImageJ (n = 4 samples per group, experiment repeated multiple times). (E) To assess cell death, infected monolayers and controls were stained for cell death marker Sytox (yellow) and Dapi (blue) for nuclei, while bacteria were shown in red (scale bar = 10 µm and 5 µm respectively). Quantification was done with ImageJ software by calculating the area of Sytox staining relative to the area of Dapi staining (n = 3 samples per group, experiment repeated multiple times). The data show mean ± SEM, * adjusted $p \leq 0.05$, ** adjusted $p \leq 0.01$, *** adjusted $p \leq 0.001$. Significant differences were observed by using by one-way ANOVA with Dunnett´s correction or two-way ANOVA with Šídák's multiple comparisons test.

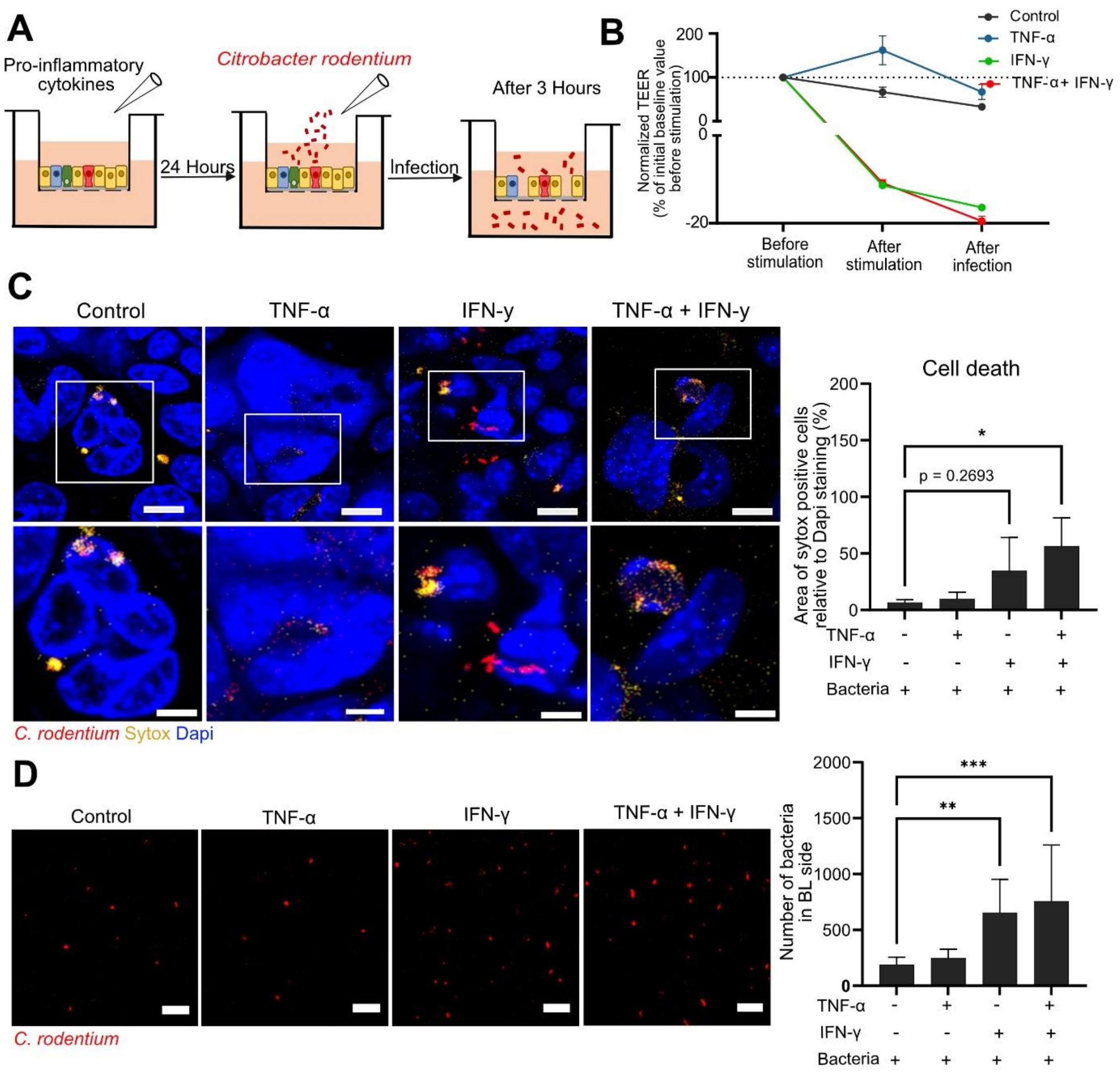

Supplementary Figure 6. *Citrobacter rodentium* translocates faster across the monolayer upon pre-treatment with pro-inflammatory cytokines. (A) Colon monolayers were treated

with pro-inflammatory cytokines for 24h prior to the infection with *C. rodentium* for 3h. (B) Barrier integrity was assessed by TEER measurements ($n \geq 3$ samples per group, experiment repeated multiple times). (C) To investigate cell death, infected monolayers and controls were stained for Sytox (yellow), as well as Dapi (blue) for nuclei, while bacteria were shown in red (scale bar = 10 µm and 5 µm respectively). Quantification was done with ImageJ software by calculating the area of Sytox staining relative to the area of Dapi staining ($n \geq 3$ samples per group, experiment repeated multiple times). (D) The number of bacteria in the basolateral side (BL, scale bar = 20 µm) was quantified by ImageJ ($n \geq 11$ samples per group pooled from 3 independent experiments). Graphs show the mean ± SEM, * adjusted $p \leq 0.05$, ** adjusted $p \leq 0.01$, *** adjusted $p \leq 0.001$. Significant differences were observed by using by one-way ANOVA with Dunnett´s correction or two-way ANOVA with Šídák's multiple comparisons test.

Legends of Supplementary Movies

Supplementary Movie 1. Establishment of colon epithelial monolayer model related to Figure 2. Immunofluorescence staining showing tight junction protein ZO-1 (Red) in fully differentiated colon monolayer, as well as Dapi, blue). 3D volume-view Z-stack confocal imaging analysed with ImageJ Software.

Supplementary Movie 2. Infection model of colon epithelial monolayer with Salmonella Typhimurium related to Figure 6. Immunofluorescence staining of tight junction protein ZO-1 (Red) as well as Nuclei (Dapi, blue) in fully differentiated colon monolayer infected with Salmonella Typhimurium (Green). The videos show 3D volume-view Z-stack confocal imaging analysed with ImageJ Software.
